# The Full Spectrum of Infective Endocarditis: Case Report and Review

**DOI:** 10.1155/2019/7257401

**Published:** 2019-01-10

**Authors:** Aniket S. Rali, Mejalli Al-Kofahi, Nilay Patel, Benjamin Wiele, Zubair Shah, Jayant Nath

**Affiliations:** ^1^Department of Cardiovascular Diseases, University of Kansas Medical Center, Kansas City, KS, USA; ^2^Department of Internal Medicine, University of Kansas Medical Center, Kansas City, KS, USA

## Abstract

Over the past five decades, the incidence of intravenous drug use- (IVDU-) associated infective endocarditis (IE) has been on the rise in North America. Classically, IVDU has been thought to affect right-sided valves. However, in recent times a more variable presentation of IVDU-associated IE has been reported. Here, we report a case of a patient with a known history of IVDU who presented with clinical symptoms concerning for right- as well as left-sided endocarditis. In addition, we also discuss what should be considered adequate evaluation for patients with suspected endocarditis, and more specifically, what should be the role of transesophageal echocardiography in patients with IE noted on transthoracic echocardiography.

## 1. Introduction

Extended intravenous drug use (IVDU) is a known risk factor for infective endocarditis (IE) [1]. Over the last five decades, the incidence of IE-related hospitalizations in North America has continued to increase with increasing IVDU. *Staphylococcus aureus* is the most common causative microorganism [2, 3]. Classically, IVDU has been thought to be the main cause of right-sided valvular IE. However, the incidence of specific valvular site involvement in patients with a history of IVDU is reported to be variable [3, 4]. A prospective, randomized clinical trial noted a higher incidence of right-sided lesions with IVDU. Two out of twenty patients had bilateral involvement [5]. In a sample population of patients with a history of IVDU who suffered death from IE, 16% were noted to have bilateral vegetations [6]. Another retrospective cohort study showed that left-sided lesions were more common than right-sided ones and that concurrent involvement was rare [7]. Older studies reported equal frequencies of right- and left-sided lesions [8].

Here, we report a case of a patient with a known history of IVDU who presented with clinical symptoms concerning for right- as well as left-sided endocarditis.

## 2. Case Report

A 56-year-old male presented with a 3-day history of altered mental status and weakness. His past medical history was significant for long-standing IVDU, chronic purulent cellulitis of bilateral lower extremities, osteomyelitis of bilateral tibiae, latent tuberculosis treated eleven years prior to presentation, and previously treated hepatitis C infection. The current hospitalization was his second within eight months, as he was previously hospitalized for methicillin-sensitive *Staphylococcus aureus* (MSSA) bacteremia due to cellulitis and osteomyelitis attributed to extensive ongoing intravenous drug injections through lower extremity veins. A transthoracic echocardiogram (TTE) performed during that hospitalization was negative for endocarditis.

During the current admission, the patient's Glasgow coma scale was 13 on presentation. Physical examination was limited by the patient's inability to cooperate, but the patient was noted to have left lower quadrant abdominal tenderness, bilateral lower extremity and right upper extremity wounds, and a large tender sacrocoxal erythematous ulcerated lesion. Presenting vital signs included a blood pressure of 140/79 mm Hg, temperature of 36.4 Celsius, heart rate of 114 beats per minute, respiratory rate of 28 breaths per minute, and oxygen saturation of 95% on 3 liters of supplemental oxygen. Laboratory studies were concerning for leukocytosis of 25.6 K/*μ*L (4.5–11.0 K/*μ*L), hemoglobin of 5.7 gm/dL (13.5–16.5 gm/dL), and platelet count of 129 K/*μ*L (150–400 K/*μ*L). Iron studies were suggestive of anemia of chronic inflammation. Other laboratory abnormalities included serum creatinine of 1.25 mg/dL (0.4–1.24 mg/dL), serum sodium of 127 mmol/L (137–147 mmol/L), and albumin of 2.0 g/dL (3.5–5.0 g/dL). Creatine kinase was 1288 U/L (35–232 U/L), lactic acid was 3.1 mmol/L (0.5–2.0 mmol/L), and troponin was 0.18 ng/mL (0–0.05 ng/mL). Blood as well as urine cultures were positive for MSSA. Furthermore, urine drug screening returned positive for cocaine and opioids. Soon after presentation, the patient developed acute hypoxic respiratory failure, hemodynamic shock, and worsening encephalopathy. He was admitted to the medical intensive care unit (MICU) for pressor support and mechanical ventilation.

Pan-computed tomography (CT) scans revealed bilateral multiple pulmonary nodular opacities, some of which were cavitary in nature concerning for multifocal pneumonia, acute hematomas in the abdominal wall musculature, and multiple subacute to chronic left cerebellar and left occipital infarcts, all concerning for septic emboli. These brain lesions were confirmed on subsequent brain MRI. Cultures from the bronchoalveolar lavage were positive for MSSA, negative fungal culture, and acid-fast stain. Further laboratory testing showed negative results in a fourth generation HIV1/2 immunoassay and in T-spot tuberculosis screening.

**Figure 1:**
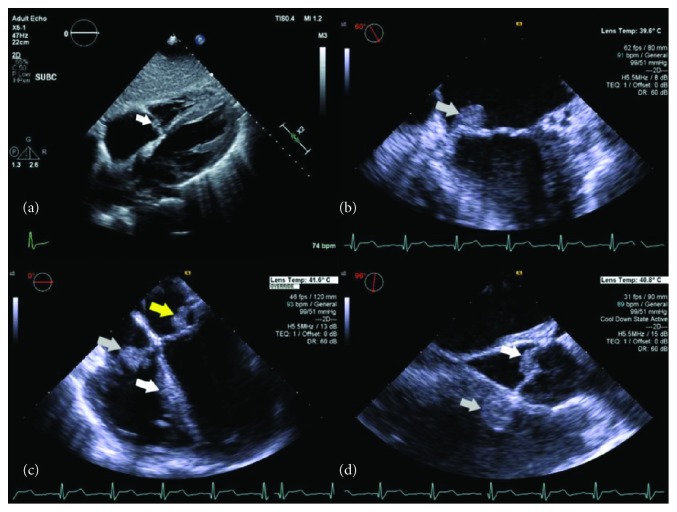
(a) Tricuspid valve vegetation on TTE (white arrow). (b) Mitral valve vegetation on TEE (grey arrow). (c) TEE showing mitral valve vegetation (grey arrow), RV outflow tract vegetation (white arrow), and tricuspid valve vegetation (yellow arrow) from left to right. (d) TEE showing RV outflow tract vegetation (grey arrow) and aortic valve vegetation (white arrow) from left to right.

A transthoracic echocardiogram (TTE) revealed a 0.5 cm mobile mass, consistent with vegetation, in the atrial aspect of the septal leaflet of the tricuspid valve without any valvular dysfunction (Figure 1(a)). Although the other valves were not well visualized on this study, the patient's left ventricular ejection function was noted to be normal. Given concerns for left-sided endocarditis, a transesophageal echocardiogram (TEE) was pursued. TEE showed vegetations on the tricuspid, mitral, and aortic valves, as well as in the right ventricular outflow tract. The tricuspid valve had a 1.0 × 1.0 cm vegetation on the anterior leaflet and a 0.5 × 0.5 cm vegetation on the septal leaflet. The mitral valve had a 1.2 × 1.1 cm vegetation on the P3 segment. There was also a 0.8 cm vegetation on the noncoronary cusp of the aortic valve with only mild aortic insufficiency. The pulmonic valve itself was without vegetations, but there was a 1.1 × 1.1 cm vegetation in the right ventricle outflow tract (RVOT) (Figure 1(b–d)).

The patient continued to receive medical care in the MICU for 2 weeks with a progressive decline in his condition. The patient was deemed to be a poor unstable surgical candidate by the cardiothoracic surgery team, and hence, the patient was transitioned to comfort care measures only after detailed discussions with the family. The patient passed away shortly thereafter from multiorgan failure. An autopsy was declined by the family.

## 3. Discussion

Our report discusses a rather unusual presentation of infective endocarditis and hence leads into the discussion of an adequate work-up for suspected endocarditis. The American Heart Association (AHA) and the European Society of Cardiology (ECS) both recommend TTE as the modality of choice for the initial evaluation of suspected IE [9, 10]. TTE is a noninvasive diagnostic modality with a reported sensitivity ranging between 44 and 70% for the detection of native valve vegetation and a sensitivity of 50% for the detection of abscesses [11, 12]. The ease of this test makes it the obvious choice for initial evaluation. However, the question begets if further evaluation is recommended if IE is already confirmed on initial TTE.

In our case, multiple bilateral vegetations leading to pulmonic and systemic septic emboli were noted on TEE, only one of which was diagnosed on initial TTE. Those bilateral vegetations ultimately guided the patient's goals of care discussion. TEE is well known to have much greater sensitivity for the detection of infective endocarditis. TEE has greater than 90% sensitivity for native valve vegetation and 90% sensitivity for paravalvular abscess. Specificity on both modalities is similar and is greater than 90% [11, 12].

To the best of our knowledge, only five previous cases have been reported where patients with IVDU had bilateral cardiac IE. All of the patients had positive *Staphylococcus aureus* bacteremia [13–16], except one who had negative blood cultures but vegetations with gram positive cocci on autopsy [17]. Among these cases, left-sided manifestations included paravalvular abscess with aortico-left atrial fistula [13], isolated mitral valve vegetations [14], aortic and mitral valve vegetations [15], extension of tricuspid vegetation through patent foramen ovale [16], and vegetation in the left ventricular outflow tract and mitral valve found on autopsy [17]. Only one prior case had more than two concurrent bilateral vegetations such as ours, with vegetations on pulmonic, tricuspid, and aortic valves with a perforation of the anterior leaflet of the mitral valve [15].

In conclusion, patients with a history of IVDU are at an increased risk for IE that could be right-sided, left-sided, or bilateral. Even though a TEE is logistically harder to obtain in the acute setting, physicians should generally be encouraged to obtain it early in the clinical course to allow for a more thorough evaluation. As was the case with our patient, this additional information can be pivotal in recommending the appropriate plan for care for patients.
